# In response to “An “outer subarachnoid space”: fact or artifact? A commentary on “Structural characterization of SLYM: a 4th meningeal membrane” fluids and barriers of the CNS (2023) 20:93 by V. Plá et al.”

**DOI:** 10.1186/s12987-024-00540-w

**Published:** 2024-06-03

**Authors:** Virginia Plá, Styliani Bitsika, Michael J. Giannetto, Antonio Ladron-de-Guevara, Daniel Gahn-Martinez, Yuki Mori, Maiken Nedergaard, Kjeld Møllgård

**Affiliations:** 1 Division of Glial Disease and Therapeutics, Center for Translational Neuromedicine, Faculty of Health and Medical Sciences, University of Copenhagen, 2200 Copenhagen, Denmark; 2 Division of Glial Disease and Therapeutics, Center for Translational Neuromedicine, University of Rochester Medical Center, Rochester, NY 14642 USA; 3 Department of Cellular and Molecular Medicine, Faculty of Health and Medical Sciences, University of Copenhagen, 2200 Copenhagen, Denmark

We thank Dr. Neuhuber for his interest in our work. We have below tried to address the comments:

Yes, we are aware that fixatives are hyperosmotic. Figure 1 in Plá et al. [1] illustrates the difference between the live and dead brain within the skull and compared to the histological section. Magnetic resonance imaging (MRI) was used to visualize both the brain and the subarachnoid spaces in live and dead mice, whereas the histological section from a decalcified whole head illustrates the effect of fixation on the brain and the meningeal membranes. The difference between the two preparations is striking, as we emphasize in the paper. The live brain fills most of the skull cavity with a small contribution from cerebrospinal fluid (CSF) in the subarachnoid space and the ventricles. After death, the CSF filled spaces are mostly gone due to swelling of the brain [2]. Yes, it is the fixation process, which is associated with a robust shrinkage of the brain what distorts the fragile meningeal membranes. 4% paraformaldehyde and other commonly used fixatives are hyperosmotic. Our measurements confirm that 4% paraformaldehyde has an osmolality that is more than fourfold greater than the physiological osmolality, of around 310 osmol (Table 1).

**Table 1:** The data represent mean ± standard error of mean (SEM) of 3 measurements of each solution

Phosphate Buffered Saline (PBS)	10% EDTA decalcification solution [3]	4% Paraformaldehyde (PFA)
301 ± 2 mOsm	773 ± 3 mOsm	1420 ± 6 mOsM

Exposure to 4% paraformaldehyde or an osmolality of > 1400 mOsm will drag fluid out of the brain, while the bony skull proportion is little affected by the fixative. The result is the creation of large artificial spaces between the skull and the brain with the consequent misplacement of the meningeal membranes. We have previously documented, using real-time imaging, the artificial transport of CSF tracers that occur when 4% paraformaldehyde hits the brain. Please see the real-time movie of the fixation artifact displayed in the perivascular spaces: Link: Supplementary video 1 [4]. It is difficult to predict how the fragile and loosely connected meningeal membranes will be displaced during the fixation, and their displacement will likely differ between animals and between regions. Once the proteins have been stiffened by the fixative crosslinking, it is unlikely that any major movements of the meningeal membranes will occur. Thus, exposing the fixed tissue to hyperosmotic solutions required for decalcification after fixation with 4% PFA is not expected to result in further misplacement of the meningeal membrane.

One important point that we want to emphasize is that all known histological descriptions of the meningeal layers are based on fixed tissue exposed to artificial displacements of the meningeal membranes. That includes the definition of the arachnoid barrier layer and the reticular/inner arachnoid layer. The description of their cellular composition and the close apposition of the layers documented in ultrastructural preparations is based on the examination of heavily fixed material and may, as such, represent a fixation artifact. Our previous examination of the meningeal membranes included an in vivo analysis of SLYM in live mice (Fig. 2) [3]. This analysis documented that the subarachnoid space is subdivided into two compartments. We chose in the follow-up analysis to include immunohistochemical identification of the newly proposed arachnoid barrier layer (DPP4) in freshly resected brain and the surrounding skull. This ex vivo analysis documents the SLYM barrier function by demonstrating that CSF tracers remained in the perivascular spaces and cisterns after extraction of the unfixed brain, while the arachnoid barrier layer remained attached to dura (Fig. 2) [1].

We have already described that SLYM is a thick 2–3 cell layer membrane at the ventral surface of the brain. At the dorsal brain surface, SLYM often fuses with the arachnoid barrier layer and is most often just a single cell layer [3]. The primary role of SLYM is to create the pial perivascular spaces thus separating the pial peri-arterial and peri-venous spaces to ensure unidirectional glymphatic transport. A recent publication used magnetic resonance imaging (MRI) [5] to show that intrathecally delivered contrast agent preferentially enters the brain along the periarterial spaces rather than mixing with the larger subarachnoid space, demonstrating a compartmentalization of the subarachnoid space in human resampling our observations in mouse brain [6].

There is no reason to expect that the meningeal membranes are structurally and organizationally similar in different parts of the CNS and we will not be surprised if SLYM is fused with the arachnoid barrier layers in the vertebrate cavity. It is thus imperative to specify which region is studied when describing the meningeal membranes.

## Data Availability

Not applicable.
